# Volumetric modulated arc therapy (VMAT): a review of clinical outcomes—what is the clinical evidence for the most effective implementation?

**DOI:** 10.1259/bjr.20201289

**Published:** 2022-06-10

**Authors:** Sherisse Ornella Hunte, Catharine H Clark, Nikolay Zyuzikov, Andrew Nisbet

**Affiliations:** 1 Radiotherapy Department, Cancer Centre of Trinidad and Tobago, St James, Trinidad and Tobago; 2 University of the West Indies, St. Augustine, Trinidad & Tobago; 3 Radiotherapy Physics, UCLH NHS Foundation Trust, London, UK; 4 Metrology for Medical Physics National Physical Laboratory, Teddington, UK; 5 Department of Medical Physics & Biomedical Engineering, University College London, London, UK

## Abstract

Modern conformal radiation therapy using techniques such as modulation, image guidance and motion management have changed the face of radiotherapy today offering superior conformity, efficiency, and reproducibility to clinics worldwide. This review assesses the impact of these advanced radiotherapy techniques on patient toxicity and survival rates reported from January 2017 to September 2020. The main aims are to establish if dosimetric and efficiency gains correlate with improved survival and reduced toxicities and to answer the question ‘What is the clinical evidence for the most effective implementation of VMAT?’. Compared with 3DCRT, improvements have been reported with VMAT in prostate, locally advanced cervical carcinoma and various head and neck applications, leading to the shift in technology to VMAT. Other sites such as thoracic neoplasms and nasopharyngeal carcinomas have observed some improvement with VMAT although not in line with improved dosimetric measures, and the burden of toxicity and the incidence of cancer related deaths remain high, signaling the need to further mitigate toxicity and increase survival. As technological advancement continues, large randomised long-term clinical trials are required to determine the way-forward and offer site-specific recommendations. These studies are usually expensive and time consuming, therefore utilising pooled real-world data in a prospective nature can be an alternative solution to comprehensively assess the efficacy of modern radiotherapy techniques.

## Introduction

Cancer is the second leading cause of death globally^1,2^ and a major public health concern. For over a century, radiotherapy used alone or in combination with other treatment modalities such as chemotherapy or surgery, has been proven effective for the treatment and management of cancer.^3^ Owing to the critical role of radiotherapy in the treatment of cancer, advances in radiotherapy techniques are likely to have major clinical impact and necessitate review of optimum evidence-based practice.

Modern radiation therapy techniques employ modulated photon (Intensity Modulated Radiation Therapy—IMRT, Volumetric Modulated Arc Therapy—VMAT) or particle (Intensity Modulated Proton Therapy—IMPT) beams and the dosimetric gain over 3D conformal radiotherapy (3DCRT) has been widely studied.^4^ Published surveys^5^ and reviews suggest a shift in usage from 3DCRT to VMAT^6^ specifically, combined with varying dose fractionation schemes [hypofractionation, Stereotactic Body Radiation Therapy (SBRT) and Simultaneous Integrated Boost (SIB)]. Additionally, the effect of image guidance (Image Guided Radiation Therapy, IGRT) and motion management systems on dose delivery, target positioning accuracy and reproducibility, warrants the assessment of collective clinical impact of these practices with modulated therapies.

Unlike fixed-field IMRT, VMAT allows simultaneous motion of gantry, MLC and dose rate using dynamic modulated arcs, resulting in increased conformality and enhanced sparing of the critical structures near the target.^7^ Techniques of VMAT are diverse and can employ flattening filter free (FFF) beams, standard, tangential (t-VMAT) or restricted angles (R-VMAT). The application of IMRT clinical trial outcomes^8^ to VMAT is proof of increased VMAT implementation.

Some authors consider VMAT as a type of IMRT, and since its introduction^4^ the initial divide in the literature’s nomenclature has blurred considerably in recent years, particularly with the advent of comparative proton studies.

IMPT utilises proton pencil beams which produce distinct dose distributions when compared to photons due to the characteristic Bragg peak, resulting in maximum dose deposition at a finite tissue depth followed by a sharp dose fall-off with no exit dose.^9^ Dosimetric studies suggest there may be advantages in the use of protons over photons with findings of normal tissue sparing and improved target conformity.^9–13^

Previously, there have been two review papers on the clinical use of VMAT and its outcomes, one assessing VMAT at the start of its implementation (2000–2010)^14^ and the other looking at the clinical outcomes of its implementation (2009–2016).^4^ Both noted increases in the global usage and clinical implementation of VMAT with many publications tailored to planning and feasibility studies; however, clinical outcome studies were emerging but scarce and reporting only acute toxicities.

This paper seeks to review the impact of modern radiotherapy techniques and treatment schemes on patient clinical outcomes for seven clinical sites during 2017–2021 and to establish if improved survival and reduced toxicities relate to dosimetric and efficiency gains. In analyzing the available literature on reported clinical outcomes where VMAT has been employed this review seeks to answer the question ‘What is the clinical evidence for the most effective implementation of VMAT?’

## Methods

This analysis strictly followed the guidelines of the Preferred Reporting Items for Systematic Reviews and Meta-Analyses (PRISMA) statement.^15^ The search engines used were the National Library of Medicine (PubMed/Medline) and *The British Journal of Radiology* (BJR) database. These identified articles from January 2017 to October 2021 which recorded clinical outcomes post radiotherapy using the keywords “radiotherapy, intensity-modulated” OR “VMAT” OR “arc radiotherapy” AND “treatment outcomes” OR “clinical trials” OR “evidence-based” OR “clinical outcomes.”.

### Inclusion and exclusion criteria

Publications were selected for inclusion if they were published within the above timeframe, English language only, full text articles which reported clinical outcomes (survival and toxicities) after modern radiotherapy schemes.

Exclusion criteria included any case reports, comment abstracts, dosimetric only studies, wrong technique (chemotherapy, tomotherapy, carbon ion therapy etc.) and whose main aim does not assess the treatment outcomes of VMAT.

## Results

**Figure 1.**
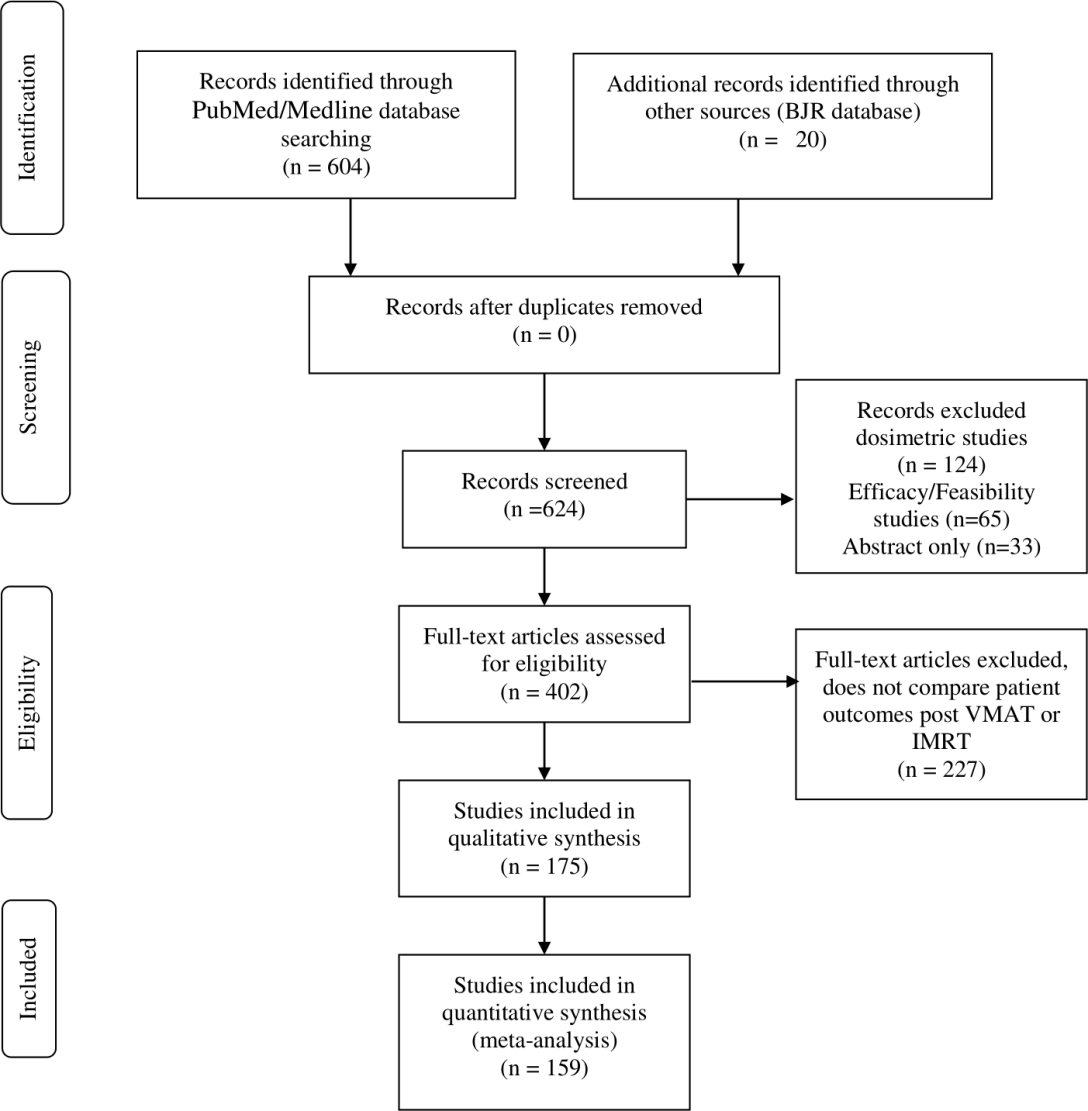
PRISMA flow diagram. IMRT, intensity modulated radiation therapy; VMAT, volumetric modulated arc therapy.

The PubMed search yielded 604 articles while searches through the BJR database identified 20 articles. After title and abstract examination for relevance and removal of publications which are present in the exclusion criteria, 175 publications remained and are included in this review (Figure 1). These assessed papers involved; retrospective studies; prospective studies and review papers.

### Prostate

The management of prostate cancer can utilise radiotherapy (photons, protons, or brachytherapy) surgery, or active surveillance.^16^ Advances in photon therapy resulted in extensive publications on dosimetric efficacy for prostate cancer and even led to the establishment of radiotherapy guidelines. The European Association of Urology (EAU) now recommends either dose escalated IMRT or VMAT as standard therapy for prostate carcinoma, due to reduced toxicity compared to 3DCRT.^16^ VMAT has been widely accepted and may be considered as first choice for radiotherapy prostate treatments due to significant reduction of rectal volume doses and improved efficiency due to reduction of MUs for some models of treatment units.^17^

Clinical implementation and recommendations^16^ are currently present without substantial empirical data from well-designed perspective clinical benefit studies.^17^ Nonetheless, recently published clinical outcome studies assessed the impact of IMRT and VMAT along with fractionation schemes and escalated doses (SIB, hypofractionation, SBRT) and image guidance techniques (IGRT) on toxicity and survival.

#### Hypofractionated schemes

Treatment fractionation has several known benefits: repair of sublethal damage to normal tissue, reoxygenation of hypoxic tumour cells, and redistribution of tumour cells to radiosensitive phases of the cell cycle.^18^ Hypofractionated radiotherapy delivers larger than 2Gy-Fraction daily doses resulting in fewer total fractions during radiotherapy.^19^ For cases of prostate cancer, hypofractionation regimens are guided by the staging. Current recommendations propose ultrahypofractionation for low to intermediate risk and moderate hypofractionation regimens for high risk.^20^ The American Society of Radiation Oncology (ASTRO) defines SBRT as “an external beam radiation therapy (EBRT) method used to precisely deliver a high dose of radiation to an extracranial target within the body, using either a single dose or a small number of fractions”^21^ and a few studies investigate its use in prostate cancer therapy.^22,23^

A recent study^24^ comparing hypofractionation (70 Gy in 28 F) *vs* conventional fractionation (80 Gy in 40 F) utilising VMAT as the treatment technique reported no significant difference in biochemical relapse-free survival (BRFS) between the groups (94.6% *vs* 95% respectively (*p* = 0.704)), and therefore support the use of hypofractionated regimes for localised prostate cancer therapy. Another study by Vassis et al^25^ also assessed hypofractionation (60 Gy in 20 F) against conventional fractionation (78 Gy in 39 F) utilising VMAT and reported hypofractionated radiotherapy schemes produce no significant difference in freedom from biochemical failure (FFBF) and late toxicities; however, significant reduction in proctitis and urinary frequency was observed. A third study^26^ of 206 males treated with step-and shoot IMRT concluded a hypofractionated regimen of 72 Gy in 2.4 Gy fractions increased the biologically effective radiation dose to the prostate, providing better control than conventionally fractionated of 75.6 Gy in 1.8 Gy fractions. Additionally, hypofractionated schemes reduced treatment duration (8.4–6 weeks) and did not correlate to increased late urinary toxicity incidence.^26^ A non-significant increase in rectal bleeding was observed, however, all cases resolved with treatment^26^ thus concluding that this regime is safe and effective.^27^ In fact, in the United Kingdom, hypofractionated radiation therapy (60 Gy in 20 Fractions) has been recommended as the new standard of care for localised prostate cases^8^ and these results guide fractionation in VMAT.

In 2017, a publication by Haque et al^28^ highlighted the lack of completed Phase III randomised trials comparing outcomes of prostate cancer patients treated with conventional fractionation to SBRT. Though consensus in prescription has not been concluded, studies reported good clinical outcomes for low-risk disease for a SBRT scheme of 35–36.25 Gy in 5 daily fractions^29–32^ however, its effectiveness in high or intermediate risk disease is less clear. A study in Philadelphia assessing 263 localised prostate cancer patients found no difference in 5-year FFBF between matched SBRT and conventionally fractionated IMRT groups and no significant difference in toxicity, concluding SBRT can be a suitable alternative treatment for patients with prostate cancer.^22^

Dosimetric comparison of proton- and photon-based hypofractionated SBRT by Goddard et al^23^ concluded when Hounsfield unit (HU) uncertainties were not addressed, IMPT and VMAT treatment plans were comparable for target coverage, conformity and OAR sparing, with proton-based plans reducing OAR dose more than VMAT. However, when HU uncertainty is considered, VMAT surpasses IMPT in terms of target conformity, and OAR sparing^23^ Additionally, a recent clinical outcome study comparing IMRT, and proton beam therapy concluded that no significant difference in biochemical failure, local failure, regional failure and distant failure was seen with these techniques.^33^ Therefore, VMAT still has a role in the future of prostate radiotherapy and further prospective studies are required to recommend another treatment modality.

#### IMRT vs VMAT

A study in 2017 of patients treated with SIB-IMRT and SIB-VMAT to the whole pelvis recorded no significant difference in the rate of acute genitourinary (GU) or gastrointestinal (GI) toxicities and no reported late Grade III toxicity of GI and GU except for rectal toxicity between the two groups.^34^ The recommendation was therefore that dose escalation using SIB-IMRT or VMAT with daily CBCT will reduce radiation toxicity to the bladder and rectum.^34^ A year later, Tondel et al studied the use of daily CBCT *vs* weekly orthogonal images on a 250 patient cohort receiving 3DCRT using field-in-field technique.^17^ Though Tøndel observed daily CBCT verification significantly reduced rectal irradiation, this gain was not translated into a reduction of acute side-effects.^17^ A similar study as Tøndel using VMAT may produce more promising clinical outcomes.

Randomised clinical outcome studies are emerging^35,36^ for locally advanced high-risk carcinoma of the prostate and conclude prostate and pelvic lymph node IMRT is safe. Although higher Grade II toxicities are observed when compared with prostate only studies^36^ low levels of GI and GU toxicity scores from physician and patient reported assessments were achieved 24 months after treatment.^35^ Whilst regional nodal irradiation provides a survival advantage to patients with localised high-risk breast cancer; studies have not concluded whether the same effect is seen in prostate cancer.^35^

Owing to the time and financial implications of randomised clinical outcome studies, currently the literature has described IMRT findings more than VMAT. However, VMAT has been used to assess fractionation^25,37,38^ and image guidance^34,39^ implying that it has been widely adopted as the gold-standard for prostate radiotherapy.

#### Anorectal

Colorectal cancer (CRC) is the third most common cause of cancer-related death worldwide^40^ and chemoradiotherapy is the current standard of practice^41^ owing to works of Ajani et al.^42^ Although historically the radiation prescription with 3DCRT was conservative, toxicity incidence was high and often required extended mid-treatment breaks resulting in reduced oncological outcomes and substantial late pelvic radiation morbidity.^43^

Current studies advocate for the use of IMRT and VMAT over 3DCRT as there are several benefits: anal sphincter sparing^44^; increased conformity and homogeneity^45^; toxicity reduction and improved clinical outcomes (Table 1).^50^ In 2017, Muirhead et al assessed the implementation of IMRT (step-and-shoot IMRT, VMAT and tomotherapy) in the UK for anal cancer and concluded that although not universal, these techniques were gaining frequency in the UK.^43^ This national audit also observed a small improvement in Grade III/IV toxicity, though statistically insignificant due to the small cohort of patients, incidence of GI (specifically diarrhoea) and haematological Grade III/IV toxicity differed between IMRT and 3DCRT with sequential boost.^43^

**Table 1.** Toxicity and survival rates for anorectal cancer patients from five retrospective studies

[ref]	Period of study	N	Prescription	RT technique	Median follow-up	Outcomes- toxicity	Outcomes- Survival
^46^	09/2014– 08/2016	11	cT2N0 Prescription: 50.4 Gy/28 to PTV 42 Gy/28 to the elective nodal PTV cT3–T4/N0–N3 59.4 Gy/33F- 61.2 Gy/34F to PTV Clinical nodes 50.4 Gy/30F if ≤3 cm or 54 Gy/30F if >3 cm Elective nodal PTV45 Gy/30F	No comparison- all cases treated with VMAT-two coplanar arcs of 360° and received concurrent chemotherapy MMC (12 mg/m^2^)	12 months (6–37 months)	Skin G3 (27.3%) G2 45.5% GI G3 18.2% G2 45.5% G3 18.2% (required hospital admission) GU G2 27.3% Haematologic G2 anaemia (18.2%), neutropaenia (27.3%), thrombocytopaenia (27.3%)	one-year OS 89% 3 year OS 71%; one- and 3 year PFS were both 75%; 2 year CFS was 68%
^47^	02/2011–04/2016	21	Phase I 39.6 Gy, 1.8 Gy/fraction Phase II 14.4 Gy up to a total dose of 54 Gy (*n* = 4) 19.8 up to 59.4 Gy (*n* = 15)	No comparison- all patients were treated with 2 full arc 6 MV VMAT plans	35.5 months (3–71 months)	Skin acute G3 (one pt) G2(47.6%); G1(38%); G0 (two pts) GU toxicity G1(47.6%) G0 (52.4%) GI toxicity Acute G3 (one pt) G2 (9.5%) G1(47.6%) G0(42.8%)	2 year OS 91% 2 year DFS 73% 2 year LC 81%
^48^	09/2007 −03/2015	155	Phase I 36 Gy (1.8 Gy/ Fraction) Phase II 23.4 Gy (1.8 Gy/Fraction)	IMRT (*n* = 39) or VMAT (*n* = 15), or HT (*n* = 97) Phase II IMRT (*n* = 16, until 2011), VMAT (*n* = 17), HT (*n* = 61) or 3D-conformal EBRT (CRT, *n* = 61)	38 months (12–51 months)	Skin Acute G3 (22%) No late Grade III cutaneous toxicity GI Toxicity late G3 (3/137 patients (anal incontinence)	4 year LC 82%; 4 years OS 82%; 4 year CSS was 90%
^49^	05/2006 −01/2015.	172	Whole pelvis at a dose of 45 Gy/ 25F	Image guided IMRT (*n* = 45) 3DCRT (*n* = 99)	53 months (range, 18–95 months) 3DCRT group 43 months (range, 17–69 months) IG-IMRT group.	Overall acute G3 or G4 toxicity IG-IMRT (8.9%) *vs* 3DCRT (20.2%) *p* = 0.042 GI G0-2 IG-IMRT(93.3%) 3DCRT (84.8%) G3/4 IG-IMRT (9.7%) *vs* 3DCRT (14.1%) *p* = 0.039	4 year OS 81.6% IMRT and 67.9% 3DCRT (*p* = 0.12 4 year DFS 53.8% IMRT and 51.8% 3DCRT(*p* = 0.51, 4-yer LFFS 88% IMRT and 75.1% 3DCRT(*p* = 0.031 4-year- DFFS 64.5% IMRT and 62% 3DCRT (*p* = 0.61)
^50^	11/2011–11/2013	15	Phase I SIB: 37.5 Gy/25F to PTV1 and 45 Gy/25F to PTV2 T2 disease 54 Gy/5F PTV boost T3–T4 disease 59 Gy/7 F Involved nodes < 5 cm 54 Gy nodes > 5 cm 59 Gy.	Sliding window IMRT (*n* = 7) VMAT (*n* = 8)	26 months (13–42 months)	Skin Acute G3 -Radiation Dermatitis (27%) GI Nausea (1.3%) Diarrhoea (13.3%) Haematologic Neutropaenia G3 (6.6%)	Three-year CFS and DFS rates were both 86% 3 year OS rate was 88%

CGS, cancer free survival; CSS, cancer-specific survival; 3DCRT, 3D conformal radiotherapy; DFS, disease free survival.

Terms: ; 3DCRT, 3D conformal radiotherapy; CFS, cancer free survival; CSS, cancer-specific survival; DFS, disease free survival; DFFS, distant failure-free survival; EBRT, external beam radiation therapy; GU, genitourinary; IMRT, intensity modulated radiation therapy; LFFS, local failure–free survival; OS, overall survival; PTV, planning target volume; RT, radiation therapy; VMAT, volumetric modulated arc therapy.

Additional studies reported similar findings to Muirhead et al reporting IMRT^41^ and VMAT alongside IGRT^46–49^ reduce acute GI and haematological toxicity and increase overall and 5-year DFS. Of note, statistically significant findings reported patients treated with fixed-gantry IMRT delivered with a sliding window technique presented a significantly higher risk of acute Grade III (or more) toxicity compared to those treated with VMAT or helical tomotherapy (38.5% *vs* 15.3%, *p* = 0.049).^48^ Toxicity and survival rates for anorectal cancer patients from five retrospective trials^46–50^ are tabulated in Table 1. These report 1- to 3-year overall survival of approximately 90% is achievable with low G3 toxicity levels. These publications confirmed the safety and efficacy of photon modulated therapies and recommend the adoption of VMAT alongside IGRT as the standard of care for anorectal cancer.

Physician-weighted and patient reported outcomes (PROs) represent a critical aspect of toxicity evaluation. An extension of the UK nationwide study conducted by Gilbert et al reported high overall 1-year oncological outcomes for overall, disease-free and colostomy-free survival consistent with the reported prospective and randomised studies of IMRT in anal cancer.^51^ The 1-year PRO toxicity data are consistent with centre reported data and suggest IMRT techniques (VMAT, tomotherapy and static IMRT) reduce bowel toxicity and male sexual dysfunction. Despite the improvement with IMRT techniques acute GI and hematological toxicity^52^ should be further reduced to minimise unplanned treatment breaks and hospitalisation.^41^ There is a clear need for further optimisation and development of planning techniques to reduce OAR dose^52^ combined with randomised prospective studies with extended follow-up to further validate the observations published and determine the durability of the findings. The usefulness of VMAT-SBRT for lymph-node recurrent cases of CRC was also studied by Franzese et al and the efficacy for local disease control confirmed.^53^

Planning studies have shown that proton therapy could significantly reduce the dose to the OARs especially pelvic bone marrow;^54^ however to date, no published clinical trial is present in the literature, so inferences can only be made from dosimetric outcomes. An ongoing clinical trial will provide answers to the usefulness of proton therapy with CRC in the next 5 years.^12^

### Gynaecological

Cervical cancer has the second highest incidence among females and is the third leading cause of cancer-related death among females worldwide.^1^ Owing to the well-established nature of VMAT and IMRT, current literature uses these techniques to assess fractionation schemes (SIB and hypofractionation) or compare with other techniques such as brachytherapy if not feasible^55^ and long-term studies compare IMRT and VMAT to 3DCRT.^56,57^

Cases of locally advanced cervical cancer (LACC) respond favourably to VMAT with low haematologic toxicity incidence and promising survival rates (Table 2).^58^ Authors even suggest that SIB-VMAT can be an effective treatment technique for irradiation of LACC where brachytherapy cannot be facilitated.^55,62^ The combined use of image guidance and VMAT (IG-VMAT) in patients with LACC reported low haematologic toxicity and promising survival rates.^58^ Current literature recommends the adoption of hypofractionated schemes, image guidance protocols and simultaneous integrated boost (SIB-VMAT) towards the enhanced management of LACC.^63^

**Table 2.** Toxicity and survival rates for cervical cancer patients for retrospective and prospective study designs

[ref]	Study design	Period of study	N	Prescription	RT technique	Median follow-up	Outcomes- Toxicity	Outcomes- Survival
^58^	Retrospective	01/2013–12/2014	18	PTV 50.4 Gy in 28 F	VMAT:2 6 MV coplanar mono-isocentric arcs	30.5 months (IQR: 13–36.25 months).	Acute haematologic toxicity Haemoglobin G0: 23.5% G1: 17.7% G2: 58.8% TLC G0: 47% G1: 35.5% G2: 17.7% Platelets G0: 88.2% G1: 5.9% G2: 5.9%	OS 72.2% (95%CI: 62.1–80.5%) DFS 2 year DFS was 63.3% (95% CI: 52.8–72.4%)
^59^	Retrospective	12/2010–05/2017 IMRT: 12/2010–09/2012 VMAT: 05/2013–05/2017	398	Phase I: whole pelvis VMAT or IMRT 45/25F or 50 Gy/28F 25–28 Phase II: Lymph node boost up to 60–70 Gy	IMRT (*n* = 67) VMAT (*n* = 331)	IMRT 35.07 (range, 4.80–90.37) months VMAT 25.47 (range, 0.93–58.93) months	VMAT group, the incidence of Grade 3 and four acute anaemia/erythropenia were 3.6 and 0.9%. acute Grade 3 and 4 leukopenia were 8.5 and 0.6%	IMRT 3 year OS: 76.2% 3 year DFS: 76.4% 3 year LC: 83.1% 3 year DMFS: 86.1% VMAT 3 year OS 80.5% 3 year DFS 65.4% 3 year LC 88.7% 3 year DMFS 78.1%. CCRT *vs* non-CCRT 3 year OS in the CCRT 84.8vs 65.4% non-CCRT (*p* = 0.005)
^57^	Multicentre randomised control trial	11/2012–08/2015	278	Phase I 45 Gy/25F or 50.4 Gy/28F	IMRT- (inverse planning approaches: IMRT, VMAT, and tomotherapy) (*n* = 129) 3DCRT (Four field box technique) (*n* = 149)	Acute effects assessed during treatment	Physician Reported Outcomes No Grade 5 AE Grade 3 and 4 AEs: 16.4%(IMRT);11.0% (3DCRT) (*p* = 0.28) GI AEs Acute Grade 2 GI AEs (IMRT- 26.2% v 3DCRT- 22.1%; *p* = 0.43). Patient reported outcomes Mean decrease- EPIC urinary summary score (wk3 and 5 compared with baseline) 3 week results IMRT (26.0 [SD, 14.5] v 22.5 [SD, 11.3]; *p* = 0.04 5 week results IMRT −210.4 [SD, 17.5] v 3DCRT 25.6 [SD, 15.3]; *p* = 0.03 EPIC urinary score at 5 weeks favoured the IMRT arm (estimate, 24.59; SD, 2.19; *p* = 0.04)	Not analyzed
^60^	Prospective study	09/2011–04/2015	30	Macroscopic disease 66 Gy/30F; Pelvis 54 Gy/30F	SIB-VMAT technique two 360^0^_ arcs with 6 MV	32 months (range: 8–50 months)	Acute GI toxicity G0- 30%; G1-23%; G2-43% Late GI toxicity G0-70%; G1- 30% Acute Urinary G0-23%; G1-40%; G2-37% Late urinary G0-90%; G1-10% Acute Vaginal G0-7%; G1-63%; G2-23% Late vaginal toxicity G0-77%; G1-23% Acute Rectal toxicity G0-47%; G1-30%; G2-23% Late Rectal toxicity G0-73%; G1-20% Acute Hematologic G0-70%; G1-30% Late Hematologic G0-87%; G1-13%	3 year OS- 93% 3 year LC 80%, Clinical outcomes by stage (II *vs* III) 3 year OS Stage II- 100% 3 year OS Stage III- 85% 3 year LC (Stg II) 91% 3 year LC (Stg III) 67%
^61^	Retrospective	01/2007–12/2016	123	45 Gy/25F or 50.4 Gy/28F	3DCRT IMRT VMAT Tomotherapy	32.2 months	Not assessed	Survival rates < 70 years vs >70 years 3 year OS 63.9vs 50.6% 5 year OS 60.4vs 39.1 % 3 year CSS 68.2vs 70.9% 5 year CSS 78.2vs 82.1 % 3 year LRRFS 82.8vs 82.1% 5 year LRRFS 78.2vs 82.1% 3 year LRFS 85.3vs 82.1% 5 year LRFS 82.6vs 82.1%

Terms: 3DCRT, 3D conformal radiotherapy; AE, adverse events; CSS, cancer-specific survival; DFS, disease free survival; DMFS, distant metastasis-free survival; EPIC, expanded prostate cancer index composite; IMRT, intensity modulated radiation therapy; LRRFS, locoregional recurrence-free survival; OS, overall survival; PRO-CTCAE, the Patient-Reported Outcomes–Common Terminology Criteria for Adverse Events; TLC, total leukocyte count; VMAT, volumetric modulated arc therapy

A comparative study of IMRT and VMAT by Lin et al^59^ observed no significant difference in 3-year survival rates (OS, DFS, LC and DMFS) however, VMAT was superior to IMRT for certain toxicity incidences, including acute anaemia, chronic enterocolitis and higher cystitis, and early-stage (IA-IIA) overall survival rates. Kloop et al^57^ compared physician and patient reported outcomes for two cohorts: 3DCRT and IMRT. Klopp defined IMRT as any inverse planning technique and therefore included IMRT, VMAT and tomotherapy in the second group. Findings from this randomised study cannot be conclusive about VMAT since both tomotherapy and IMRT were also used,^57^ however, it revealed pelvic IMRT decreased impact on bowel and urinary function and quality of life (QOL) metrics. Therefore, a reduction in the decline of physical function is observed with IMRT, VMAT and tomotherapy compared with standard pelvic radiotherapy.

Though long-term follow-up is still suggested to determine the impact on late toxicity and survival rates, the literature is consistent that VMAT has been well tolerated, safe and effective and its use alongside hypofractionation and image guidance has resulted in tangible clinical evidence of reduced GI and GU toxicity and enhanced QOL.

### Breast

3DCRT has been the gold-standard for the treatment of breast carcinoma, however, results from dosimetric and efficiency studies have steered the shift to modern techniques in many centres. Due to the involuntary motion of the lungs during radiotherapy, motion management strategies namely deep inspiration breath-hold (DIBH) is encouraged alongside photon modulated techniques to minimise heart and lung doses.^64^ Owing to the increase in 10-year survival rates, it is critical to understand and minimise long-term toxicities and late cardiac events.^65–67^ For carcinoma of the breast, this means the investigation of both modulated techniques and motion management systems.

Jagsi et al^68^ compared patients treated with 3DCRT on free-breathing scans *vs* step-and-shoot IMRT on DIBH scans and concluded that IMRT with DIBH has potential benefit to preserve cardiac ejection fraction, among patients with left-sided disease with internal mammary nodal involvement. Another study^69^ reported a reduction in mean heart doses and expected years of life lost with DIBH compared to free breathing (FB). A prospective, randomised study by Choi et al^70^ compared 3DCRT (50.4 Gy in 28 F followed by 9 Gy in 5 F boost) to SIB-IMRT (50.4 Gy in 28 F to the breast and 57.4 Gy in 28 F to the tumour bed) reported no significant difference in 3 year LRRFS, DMFS, RFS and OS. However, the IMRT cohort experienced a reduction in Grade II or higher radiation dermatitis (27.8% IMRT *vs* 37.1% 3DCRT) and lower dose to the ipsilateral lung and heart (for LBreast), therefore there is promising data for the use of IMRT for early-stage breast cancer.^70^

Literature on the use of VMAT for breast radiotherapy has reported enhanced tumour coverage, increased dose homogeneity and conformity,^71^ with one main drawback, the generation of low-dose baths specifically to the contralateral breast, lung and heart which exceed that of 3DCRT.^72^ In a study by Ma et al,^73^ VMAT was associated with an increase in mean heart dose and low-dose volume to the lung, compared with 3D-CRT, possibly explaining the higher use of tangential IMRT over VMAT.

An estimation of excess absolute risk (EAR) in terms of developing a secondary cancer in three organs [contralateral breast (CB), contralateral lung (CL), ipsilateral lung (IL)] after exposure to radiation was determined by Haciislamoglu et al.^74^ They observed a significantly lower EAR risk with field in field (FiF) technique and a statistically lower secondary cancer risk compared with IMRT and VMAT.^74^ Additionally, the volume of low dose (3 Gy and 5 Gy) to normal tissue was significantly higher with IMRT and VMAT than FiF.^74^ Whole breast hypofractionated VMAT has been studied and implemented in some centres and reports of safety, efficiency and tolerated patient experience has been noted.^75^ New techniques such as tangential VMAT (t-VMAT) and tangential IMRT (t-IMRT) has been compared and found while t-VMAT produced higher target homogeneity and conformity, t-IMRT for left-sided breast carcinoma resulted in significant reduction in heart and lung doses and a greater than 40% reduction in heart and lung EAR.^71^

The optimal radiation technique to treat breast cancer can vary with patient anatomy and laterality of the breast cancer and size of the treated field. Long-term clinical trials and theoretical estimation studies are required to determine the effects of the low-dose baths seen with VMAT on the healthy tissue and secondary cancer induction. Though some dosimetric promise has been reported with proton therapy (mean heart dose < 1 Gy),^11^ the clinical impact on late cardiac toxicity of this and other modern techniques is currently unknown and a minimum of 10 years of follow-up would be required to determine the effect of modern techniques on toxicity and survival.^76^

#### Thoracic neoplasms

Stage I non-small cell lung cancer (NSCLC) can be optimally treated with surgery providing good local control and survival outcome.^77^ However, for a significant number of patients, this option is not viable due to comorbidities and as a result receive concurrent chemoradiation therapy (CCRT). Historically, CCRT corresponded to relatively poor outcomes: long-term survival 15–30%; local control 40–50%^77^ ; median survival time of 28.7 months.^78^ Radiation-induced toxicity, specifically radiation pneumonitis (RP) incidence impacts survival and QOL,^79^ and thus is a factor of interest when comparing techniques and treatment efficacy.

In recent years, the use of modern conformal techniques (IMRT, VMAT, IGRT, SBRT) have dramatically changed the treatment capability and expected clinical outcomes for NSCLC (Table 3).^86^ Though large variations in treatment plans are seen with planners of different experience levels,^87^ studies have reported increased 5-year OS rates and favourable toxicity profile with SBRT using IMRT or VMAT.^77,88^ Chi et al^88^ assessed the clinical outcomes of following particle beam therapy and SBRT and observed for both techniques incidence of severe toxicity (Grade III-V), chest wall toxicity and RP were low, and no significant difference was seen between techniques in incidence of Grade IV-V toxicity. Additionally, Liao et al 2018^78^ reported comparable incidence of RP between IMRT and passive scatter proton therapy (PSPT) and IMRT reported a slightly better, but not significant, overall survival rates (*p* = 0.297) may be produced with PSPT for NSCLC. Another study observing the effect of IMPT and concurrent chemotherapy on thoracic tumours also observed low toxicity for advanced inoperable cases; however, due to the short follow-up and non-randomised study design concluded that further randomised prospective trials are required to validate and accurately quantify the effect of IMPT use.^82^ Although it cannot be concluded that PSPT or SBRT is better for the treatment of NSCLC, we can say with confidence that these are viable options and offer improved outcomes than those seen historically.

**Table 3.** Toxicity and survival rates for thoracic neoplasms and NSCLC for retrospective and prospective study designs

[ref]	Study design	Period of study	N	Prescription- diagnosis and staging	RT technique	Median follow-up	Outcomes- toxicity	Outcomes- survival
^80^	Retrospective	09/2015–10/2018	27	60 Gy/8 F Patient cohort: Central thoracic oligometastases	SBRT- IMRT or SBRT-VMAT	11.6 months (IQR 6.5–19.4 months)	No Grade > 3 toxicities Acute Grade II toxicity-3 (11.1%) Grade II dysphagia-one case: 1 month post-SBRT resolved by 3 month Grade II radiation pneumonitis - 2 cases resolved with steroid treatment. Grade 2 late Toxicity - fatigue, One (3.7%)	1 year IFC 95.2% (95% confidence interval [CI] 86.6–100.0%) 2 year IFC 85.7% (95% CI 68.3–100.0%) 1 year PFS 42.8% (95% CI 26.1–70.1%) 2 year PFS 23.4% (95% CI 9.8–55.9%) 1 year OS 82.7% (95% CI 68.6–99.7%) 2 year OS 69.5% (95% CI 51.0–94.7%)
^81^	Retrospective	03/2011–09/2016	134	52 Gy/26F or 64 Gy/32F Diagnosis: Stage I-IV NSCLC	VMAT- 6 MV single arc or two-arc	18.6 months (range, 2–45 months)	Radiation pneumonitis Grade 0–2 (*N* = 120) Grade 3–4 (*N* = 14) Fibrosis Grade 0–2 (*N* = 122) Grade 3–4 (=12) Further toxicity classification seen in Table 2	2 year PFS 18.2% median PFS 7.6 months 2 year OS 38.4%, median survival time of 18.6 months
^77^	Retrospective	11/2007-06/2016	300	48 Gy in 12 Gy × 4 fractions. Diagnosis: Stage I NSCLC T1a: 59% (*N* = 111) T1b: 30% (*N* = 57) T2: 11% (*N* = 21) All patients were N0 and M0.	2007–2010, Patients treated with (*n* > 7) coplanar/ non-coplanar beams After 2010, all patients were treated (VMAT) two half arcs	18 months (IQR: 9–33 months)	No Grade IV or V toxicity observed Fatigue (41%; *N* = 77) Chest wall pain (10%; *N* = 19) Dyspnea (7%; *N* = 14) Radiation pneumonitis (4%; *N* = 8, including 2% of Grade 3), Dermatitis(4%;*N* = 7) Cough (3%;*N* = 6) Rib fractures (2%; *N* = 3) Esophagitis (1%; *N* = 1)	1 year OS 83% [95% CI; 78–89%] (*N* = 128) 2 year OS 65% [95% CI: 57–73%](*N* = 78) 4 years, the OS 37% [95% CI; 29–47%] (*N* = 53) Median survival 37 months. 1 year DFS 75% [95% CI: 68–81%] (*N* = 114) 2 year DFS 49% [95% CI: 42–58%] (*N* = 60) 4 year DFS 31% [95% CI: 24–41%] (*N* = 41)
^6^	Retrospective	01/2009-03/2017	3872	Diagnosis: Stage III NSCLC	3DCRT - *N* = 1178 (30.4%) IMRT *N* = 1847 (47.7%) VMAT *N* = 847 (21.9%)	Not stated	Not assessed	1 year OS 3CRT Group 74.4% (71.8–76.8); 1 year OS IMRT Group 74.4% (71.8–76.8) 1 year OS VMAT Group 77.5% (74.6–80.2) 5 years OS 3DCRT 22.4% (95% CI, 20.0–25.0) 5 year OS IMRT 23.5% (95% CI, 21.3–25.7) 5 year OS VMAT 23.9% (95% CI, 20.0–28.0)
^82^	Non-randomised trial	2012–2016	51	Not specified newly diagnosed or recurrent Stage II or III NSCLC	Multifield optimised IMPT plans three or four beams	23.0 months (range 0.9–60.1 months)	Pneumonitis G1 16% G2 14% Cardiac toxicity G1 6% G2 8% Oesophagitis G1 37% G2 43% G3 6% Radiation dermatitis G1 33% G2 31% G3 6% Pain G1 37% G2 29% Oesophageal stricture G3 2% Fatigue G1 63% G2 27% G3 2% Further analysis refer to Table 4	Median OS 33.9 months Median DFS 12.6 months 3 year LC 78.3% 3 year DFFS 51% Further analysis refer to Table 2

Terms: 3DCRT, 3D conformal radiotherapy; DFFS, distant failure-free survival; DFS, disease free survival; IFC, in field control; IMRT, intensity modulated radiation therapy; LC, Local control; NSCLC, non-small cell lung cancer; OS, overall survival; PFS, progression-free survival; RT, radiation therapy; SBRT, stereotactic body radiation therapy; VMAT, volumetric modulated arc therapy.

**Table 4.** Toxicity and survival rates for nasopharyngeal carcinoma for retrospective and prospective study designs

[ref]	Study design	Period of study	N	Prescription- diagnosis and staging	RT technique	Median follow-up	Outcomes- toxicity	Outcomes- Survival
^83^	Retrospective	10/2010-05/2014	20	SIB-VMAT three target volumes: 70 Gy/35F, 63 Gy/35F and 56 Gy/35F SEQ-VMAT two targets: 46 Gy/23F boost field 24 Gy/12F Diagnosis: nasopharyngeal cancer; clinical stage of II, III, IVA, or IVB	SIB- VMAT And SEQ-VMAT	All patients: 47 months (range 8–70) f Survivors: 51 months (range 26–70)	Haematological toxicities Grade III or higher toxicities Anaemia 4 (20%) Leukocytopaenia 12 (60%) Thrombocytopaenia1 (5%) Elevation of creatinine 1 (5%) Hyponatraemia 9 (45%) Non-haematological toxicities, Grade III or higher toxicities Mucositis 11 (55%) Dysphagia 10 (50%) Dermatitis 8 (40%)	3 year OS 85% [95% CI 69–100] 3 year PFS rates 65% (95% CI 44–86) 3 year LCR, 78% (95% CI 59–97), 3 year RCR 88% (95% CI 73–100) 3 year DMFR were 79% (95% CI 61–97)
^84^	Prospective	06/2013-08/2015	80	The prescribed doses were as follows: 68–72 Gy to the PGTVnx, 64–68 Gy to the PGTVnd, 60 Gy to the PTV1, and 54–56 Gy to the PTV2, in 30–33 fractions Staging: (i) newly diagnosed cases of primary NPC with pathological confirmation Stage I-IVB	Single or two-arc VMAT (*N* = 40) 7–9 field IMRT (*N* = 40)	29 months (range,6–48 months)	Not assessed	2 year estimated LRFS 100% VMAT and IMRT 2 year RRFS and 2 year LRRFS 97.4% VMAT 100% IMRT 2 year DMFS and DFS 90% VMAT and 95% IMRT 2 year OS rates were similar between two groups, with the 92.4% VMAT and 97.5% IMRT Local failure did not occur in both groups until the end of follow-up
^85^	Prospective	03/2013-12/2015	140	The prescribed dose and fractionation for PTV-H, PTV-M, and PTV-L were 69.96 Gy, 59.40 Gy, and 52.80 Gy in 33 fractions, respectively New diagnosis of non-distant metastasis NPC	7-field SIB-IMRT (*n* = 74) Dual arc SIB-VMAT (*n* = 66)	IMRT group46 months (range, 2 - 59 months) VMAT group 38 months (range, 12–58 months)	Not assessed	Survival rates VMAT *vs* IMRT The 3 year LRRFS 96.6% VMAT group and 91.4% IMRT 3 year DMFS 89.4% VMAT; 90.0% IMRT 3 year FFS 86.1% VMAT; 79.8% IMRT 3 year OS 87.4% VMAT 91.3% IMRT (*p* value > 0.05 Survival rates: N0 *vs* N2-3 disease 3 year LRRFS DMFS, FFS, and OS rates for N0– one were 96.9, 94.0, 88.8, and 95.7% 82.5%, 82.2%, 76.3%, and 80.5% for N2–3 **(all *p* values < 0.05)** Also, larger GTV was observed to be predictive of poorer LRRFS.

Terms: 3DCRT, 3D conformal radiotherapy; DFFS, distant failure-free survival; DFS, disease free survival; DMFR, distant metastasis free rates; DMFS, distant metastasis-free survival; FFS, failure-free survival; IMRT, intensity modulated radiation therapy; LC, Local control; LCR, local control rates; LRRFS, locoregional relapse-free survival; NSCLC, non-small cell lung cancer; OS, overall survival; PFS, progression-free survival; RT, radiation therapy; SBRT, stereotactic body radiation therapy; VMAT, volumetric modulated arc therapy.

Retrospective studies^6,77,80,89^ record improved survival and reduced RP incidence and severe toxicity with VMAT compared with 3DCRT and even IMRT and report a significant increase in use of VMAT after 2010.^6,77^ Radiotherapy management of locally advanced NSCLC has therefore improved with modern photon therapies; however, the survival rates and toxicity incidence can possibly be improved by standardising treatment planning, implementation of new techniques, increased use of motion management systems and assessment of fractionation schemes. As an example, VMAT techniques with FFF beams are potentially advantageous as they employ higher dose rates and jaw-tracking technology which may further increase conformity and OAR sparing.^81^ At the very least, it can be concluded that the use of VMAT in NSCLC and other thoracic neoplasms offers no detriment to survival rates historically seen.

### Head and neck

Radiotherapy has and continues to play a critical role in the management and treatment regime for head and neck (HNC) cancers.^9^ Historically, photon-based techniques dominated HNC treatment; however, there is a growing interest in modulated proton therapy for clinical use. One major drawback of IMPT is its high sensitivity to anatomical changes which is critical in HNC due to inter- and intrafractional motion and target volume changes with high weight loss.^9^

Toxicity profiles for patients with locally advanced head and neck cancer (LAHNC) have improved but remain significant^90^ with frequent, though reduced, reports of xerostomia.^91^ Sequential (SEQ) boost *vs* SIB for LAHNC reported comparable survival rates; however, acute toxicity incidence benefited with SEQ-IMRT.^90^ LAHNC are susceptible to locoregional recurrence and as such reirradiation was explored.^92^ Bahl et al recommended a prescription >46 Gy for inoperable recurrent tumours as < 45 Gy showed a higher incidence of progressive disease ( =0.01).^93^ Additionally, QUANTEC guidelines were released 10 years ago with much of the evidence based on conventional 3D conformal therapy. There is therefore a need for validation and prospective studies to guide the optimisation of the treatment plans generated in this era.^94^

#### Nasopharyngeal cancer

For nasopharyngeal cancers (NPCs), the advent of IMRT facilitated reduction in dose to OARs and improved target homogeneity.^95^ A few randomised controlled trials have assessed the clinical benefit of IMRT compared to 3DCRT.^96–98^ Studies comparing IMRT and VMAT in NPC have shown the variation in plan conformity, coverage and homogeneity are marginal.^84^ Additionally, clinical outcomes of tumour control, survival, and changes in QOL are comparative between IMRT or VMAT (Table 4).^83–85^

It has been shown that particle therapy (IMPT) can offer OAR sparing and target conformity with promising initial findings.^99^ However, evidence for the translation of these benefits to efficacy and toxicity are limited. It is therefore safe to say despite the enhancement in NPC management in the last 30 years, the burden of long-term toxicities, which impair QOL, is still present. Adaptive radiotherapy, IGRT and the comparison of clinical outcomes (more than 5 years) between particle and photon therapies fueled by PROs is the next step for research. Additionally, specific research geared towards the decrease in cognitive and hearing impairment is necessary to improve QOL of the survivors.

#### Oropharyngeal cancer

The average 5-year overall survival for oropharyngeal cancers is 65% with traditional radiotherapy techniques.^9^ The use of VMAT for oropharyngeal carcinoma has been published and viewed as safe and effective with increasing rates of survival and disease control.^100^ Prospective proton therapy studies^101^ have shown clinical benefits for oropharyngeal cancer with reduced rates of PEG-tube replacement, acute hospitalisation and narcotic requirements compared to VMAT. Although longer follow- up is needed to determine long-term effects, initial findings show a reduction in acute toxicity and hence improved quality of life. Owing to the sensitivity of proton beams to radiological density changes, the use of on-board imaging to confirm setup and anatomical reproducibility is mandatory^9^ alongside long-term prospective studies that can clearly quantify the clinical benefit of IMPT over IMRT and establish recommendations for safe and effective treatment.

#### Multiple brain metastases

Whole brain radiotherapy (WBRT) was the initial practice of care for multiple brain metastases^102^ and single large brain metastases with the latter also employing the use of surgical resection.^91^ Currently, there has been a shift from WBRT to SRS where possible as a 12-fold reduction in 1-year local failure is gained with the use of SRS^103^ along with increased OAR sparing, improved outcomes, and increased life expectancy.^102^

Additionally, WBRT is associated with reduced QOL from decreased neurocognitive function and increased memory loss. A Phase II RTOG 0933 clinical trial^104^ proposed employing strategies to avoid the hippocampus during WBRT (HA-WBRT) as a possible approach to mitigate these events. Some practical strategies have been published,^105^ which have been employed for cases of diffused metastases where SRS was not permissible.^106^

The commercial solutions for delivering SRS are wide ranging employing Cobalt-60 sources (Gamma Knife (GK) unit), photon-based deliveries (CyberKnife (CK)) or a gantry-based linear accelerator system with stereotactic capabilities.^107^ Historically, the use of GK platform for radiosurgery was preferred; however, treating more than five metastases with GK burdens staff resources as treatment is associated with long times (1–3 h) especially as the cobalt-60 source decayed.^108^ The use of Linac-based SRS employing FFF beams have now been extensively used as its high dose rate is more time efficient (approximately 20 min).^108^ VMAT SRS when compared to GK also improved target conformity with no significant difference between 6 MV FFF and 10 MV FFF. However, gradient index may be a more relevant parameter to study and one downfall is the increased low-dose baths compared to GK for which the clinical significance is currently unknown^108^

#### Glioblastoma multiforme (GBM)

Published literature has illustrated the dosimetric and efficiency advantages of VMAT to IMRT for GBM management in treatment time reduction, dose reduction to the brain stem, hippocampi, optic chiasm and cochleae and improved target coverage and conformity,^109–111^ therefore explaining the increased implementation of VMAT over IMRT without clinical outcome data. Sheu et al was the first to assess the clinical benefit of VMAT for GBM and deduce if dosimetric advantages translated to clinical outcomes.^108^ Toxicity was assessed and recorded weekly and an MRI with contrast taken 1-month post-RT.

No significant difference was observed in median OS (18.4 months IMRT *vs* 22 months VMAT: *p* = 0.33) and dermatological toxicities (81% alopecia; 58% erythema); however, fatigue (57%) and headaches (20%) were reported by both groups with no difference in toxicity incidence as well. Sheu et al concluded that care should be used in correlating dosimetric gain to clinical effects and centres should understand new techniques before adoption.^108^

Another study assessed the impact of chemoradiation using VMAT on survival and disease progression or tumour failure at the contralateral hippocampus (cHC) for 82 patients with GBM over 4 years (2014–2018).^112^ The median follow-up for survivors was 11.7 months (range, 3.6–39.1) with a median OS of 23.5 months (95% CI: 18.4–28.7 months) and median PFS of 9.7 months (95% CI, 7.9–11.5 months). 6- and 12-month cHC failure-free rates were high at 98.7 and 97.2% respectively and overall tumour-failure at the cHC was low with 7.3% observed at the cHC and 9.8% failure observed at a 1-cm margin to the cHC.^83^ Wee et al therefore concluded that chemoradiation using HA-VMAT produced low incidence of cHC- and cHC + 1 cm-failure, and therefore can be safe in newly diagnosed cases of GBM once this technique does not impair target coverage.^112^

Although there are several dosimetric studies of the potential impact of proton therapy for GBM, a recent publication^113^ observed that although the radiation exposure to normal tissue responsible for cognitive function was significantly less with proton therapy this did not translate to improved cognitive outcomes.

### Oesophageal

The use of radiotherapy for both resectable and unresectable oesophageal cancer is well understood and is effective and essential to its management.^114^ Numerous planning studies highlighted the superiority of IMRT over 3DCRT; however, the question of association between dosimetric gain and clinical effects remain. Xu et al reported a significant reduction in survival for patients treated with 3DCRT (*p* = 0.007) when compared to IMRT; however, the two techniques produced similar incidence of radiation pneumonitis and radiation oesophagitis.^114^ Another study highlighted by Gwynne et al^115^ assessed long-term clinical outcomes of patients treated with 3D-CRT (*n* = 413) and IMRT (*n* = 263) and reported a statistically significant increase in the risk of dying and of locoregional recurrence with 3DCRT compared to IMRT (72.6% *vs* 52.9%, *p* < 0.0001; *p* = 0.0038 respectively).

Cone beam CT (CBCT) has been proven to be reliable in pre-treatment target verification and greater setup reproducibility in the treatment of oesophageal and gastrooesophageal cancers^115,116^ and has since been mandated in the UK’s NeoSCOPE/SCOPE two trial for IGRT usage. Initially, due to concerns of the effect of the low-dose baths from IMRT and VMAT NeoScope did not allow these techniques in their earlier trials, however, owing to the benefits seen with these techniques they have been mandated to be used in the current SCOPE two trial.^117,118^

Zhao et al^13,119^ reported both the favourability of reduced toxicity (less than Grade IV) with modern techniques and concern of high LR failure. Dosimetric studies point to proton therapy for clinical improvement,^120^ however, multicentre, long-term (greater than 5 years) prospective randomised trials aimed at technique standardisation, effective comparison of the various techniques and ultimately recommendations are needed.^114^

#### Radiotherapy and pregnancy

Malignant tumours occur in 1:1000 pregnancies^121^ with breast cancer followed by gynaecological malignancies and lymphomas being the most diagnosed tumours in pregnant females.^122^ Radiotherapy during pregnancy, though not impossible, require careful considerations based on tumour location and gestational age as the foetal effects are vast: abortion, foetal death, microcephaly, and foetal malformations.^123^ Modern photon modulated techniques require specific considerations and multidisciplinary input owing to the creation of low dose-baths by IMRT or VMAT approaches and use of kV-generated images for IGRT.^122^

#### Breast radiotherapy in pregnancy

Intraoperative radiotherapy (IORT)^124^ has been identified as a satisfactory boost to the tumour bed in carcinoma of the breast. However, treatment of breast cancer with radiation poses a challenge due to the proximity of the foetus to the tumour bed. Hence, international consensus supports a gestation stage-based treatment approach for breast cancer and recommends possible post-ponement of near-term patients more than 37 weeks of gestation where the treatment can be post-poned until post-partum in near-term patients at more than 37 weeks of gestation.^122^

In 2011, the Italian European Institute of Oncology treated the first pregnant patient with electron beam intraoperative radiotherapy (ELIOT) at week 15 of gestation and an estimated dose to the foetus was 0.84 mGy. These results suggest that ELIOT can therefore be considered as a treatment option for anticipated boost therapy during the first and second trimester of pregnancy and whole breast radiotherapy post-poned until after childbirth.^122^ The inclusion of a multidisciplinary team goes without saying and as much as possible treatment should follow established guidelines for non-pregnant patients.^125^

#### Pelvic radiotherapy in pregnancy

Spontaneous abortion has been observed within 3–6 weeks of pelvic RT.^122^ Cases of cervical cancer diagnosed after the 20th week of gestation; a treatment delay can be considered in the interest of the foetus without a significant effect on the prognosis.^122^ No international consensus or recommendations have been published for pelvic RT during pregnancy as the risk of foetal defects and abortion with radiotherapy is significant.^122^

#### Lymphoma and pregnancy

Evens et al^126^ investigated the effects of chemotherapy, RT or a combination of both in Hodgkin’s lymphoma and non-Hodgkin’s lymphoma in a series of 90 pregnant females. Four cases (4.4%) utilised radiotherapy with Stage I and IIA diagnoses and dose prescription of 25–30 Gy. No spontaneous abortions, neonatal intensive care unit admission or malformations were reported therefore radiotherapy in pregnant patients with lymphoma may be feasible and modern radiotherapy techniques can be explored.

#### Oral cancer and pregnancy

The incidence of oral cancer during pregnancy is less than 2% and the treatments are as follows: surgery (56.4%), chemotherapy (12.8%), radiotherapy (28.2%), no treatment during pregnancy (23.1%).^120^ Like other clinical sites, no clear guidelines exist for the treatment of oral cancer during pregnancy^120,127^ Treatment strategies should involve careful investigation of patients staging and social history.^128^

Takahashi et al^120^ studied a 36-year old tongue cancer patient treated during pregnancy using FFF-VMAT technique. Dosimetric comparison between tomotherapy, single arc VMAT and FFF-VMAT showed significant out of field doses due to scatter from the flattening filter. FFF-VMAT attained the lowest simulated foetal dose with phantom study and was therefore selected with the following prescription: involved nodes - 66 Gy in 33 F, tumour bed and ipsilateral neck - 60 Gy in 33 F; contralateral neck - 54 Gy in 33 F. The actual foetal dose was measured using *in vivo* dosimetry and calculated to be 30 mGy and a baby was born healthy at 37 weeks.

#### Brainstem gliomas

Brainstem gliomas though rare (approximately 2% of adult gliomas) occur more in younger adults,^128^ and hence must be studied when evaluating the suitability of radiotherapy during pregnancy. Brainstem gliomas are associated with high maternal mortality,^128^ therefore treatment with surgery or radiotherapy should not be delayed. Despite the high mortality, Rosen et al observed^128^ some favourable pregnancy outcomes and, concluded that the most optimum treatment plan can be determined through *in vivo* monitoring and phantom estimation studies.

#### Oropharyngeal cancer in pregnancy

Pineda et al observed the effectiveness of 6 MV IMRT on a patient with oropharyngeal cancer treated during pregnancy and concluded that in this case radiotherapy alone provided good local control to the patient and did not result in any foetal abnormalities at birth or 18 month post-delivery.^129^ This is a promising outcome; however, care must be taken for radiotherapy delivery during pregnancy.

## Conclusion

VMAT has been widely adopted throughout many centres and many clinical sites. Proof of this adoption can be seen in its use to evaluate the effectiveness of other techniques (chemotherapy regimens, SIB, FFF, SBRT, IGRT and hypofractionation)^37–39,130–133^ and the application of outcomes in dose fractionation gained through static IMRT studies seamlessly applied to VMAT.

VMAT has surpassed 3DCRT in most sites and has been proven to be more efficient while providing increased OAR sparing, reduced toxicity, and improved survival rates. As such, authors as well as some multicentre clinical trials^117,118^ recommend the adoption of VMAT for treatment of prostate and gynaecological carcinomas as well as thoracic neoplasms—specifically NSCLC—and head and neck applications (SRS, HA-WBRT, nasopharyngeal, oropharyngeal, oesophageal and gastroesophageal carcinomas). Long survival sites such as breast have not ruled out 3DCRT through field-in-field techniques and advocate for selection of radiation technique based on patient anatomy and laterality of the breast cancer and size of the treated field. The concern of the low-dose baths seen in VMAT on healthy tissue and secondary cancer induction is especially important for breast radiotherapy and when addressed may steer the shift to VMAT from the mainstay of 3DCRT.

Although authors have and continue to report many improvements with VMAT, most study designs are retrospective in nature and assess small patient cohorts. Additionally, toxicity and survival has not fallen as theoretically expected in some cases namely thoracic neoplasms and nasopharyngeal carcinomas, therefore requiring focus into standardising treatment planning and further assessment of fractionation schemes. There is a need for more prospective, multicentre, long-term, randomised clinical trials with large patient cohorts to accurately answer our research question.

Prospective randomised clinical trials are expensive, lengthy and may have small study groups, however, the use of pooled real-world data in a prospective nature can be an alternative solution to comprehensively assess the efficacy of modern radiotherapy techniques.^91^ Collaborations between the European Organisation of Research and Treatment of Cancer and the European Society for Radiotherapy and Oncology) has produced the - E²-RADIatE (EORTC 1811 study) platform designed to collect real-world data through prospective data registries in radiotherapy.^134^This and other initiatives like this may be the direction for definitive evaluation of techniques efficacy and their impact on toxicity and survival.
